# Supplementary material for: Segregation of brain and organizer precursors is differentially regulated by Nodal signaling at blastula stage
Source: Biol Open. 2021 Feb 25;10(2):bio051797. doi: 10.1242/bio.051797 (PMC7928228; doi:10.1242/bio.051797)
Supplement: Supplementary information [file biolopen-10-051797-s1.pdf]

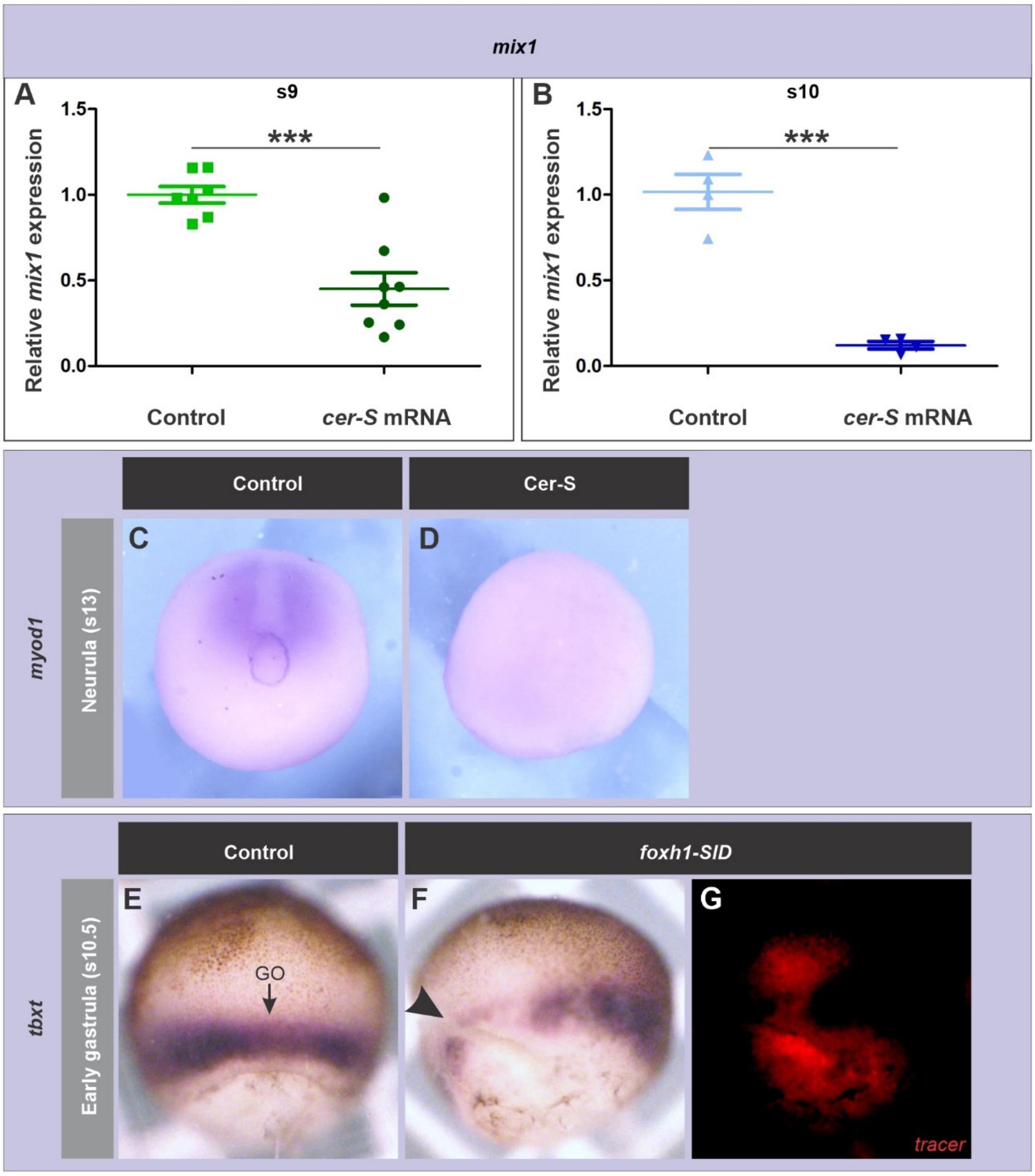

Figure S1. Control experiments testing the *cer-S* and *foxH1-SID* constructs employed in this study. (A,B) Relative expression levels of *mix1* transcripts at s9 (A) and s10 (B) in *cer-S*-injected embryos and uninjected sibling controls, analyzed by RT-qPCR. Values for each biological replicate are indicated by symbols. *Cer-S*-injected biological replicates were tested by the effect on the expression of *mix1*, a direct target of Nodal signaling (Charney et al., 2017). *mix1* expression was significantly reduced ($p<0.05$, unpaired, two-tailed t-test) in *cer-S*-injected embryos compared to uninjected sibling controls, both at s9 ($p=0.0003$) and at s10 ($p=0.0001$). Only those biological replicates that showed *mix1* expression reduced to less than 50% in relation to uninjected sibling controls were used for RT-qPCR analysis of the other markers shown in this work. (C,D) Effects of *cer-S* on the paraxial mesoderm marker *myod1.* Control neurula (C) showing *myod1* expression, which was abolished or drastically reduced in cer-S-

Biology Open • Supplementary information

injected siblings (D). (E-G) Effects of *foxh1-SID* on the expression of the pan-mesodermal marker *tbxt*. (E) Control early gastrula (s10.5) showing *tbxt* expression throughout the involuting mesoderm, including the GO. (F,G) Sibling gastrula unilaterally injected with *foxh1-SID* mRNA. The injected side is evidenced by the tracer's red fluorescence (G). *Foxh1-SID* decreased *tbxt* expression in 100% of the injected embryos (n=17, N=1), as expected. Embryos are shown in dorsal views and are siblings of those analyzed for *chrd.1* expression in one of the experiments shown in Table 2.

References for Supplementary Figure S1

Charney, R. M., Paraiso, K. D., Blitz, I. L. and Cho, K. W. Y. (2017). A gene regulatory program controlling early Xenopus mesendoderm formation: Network conservation and motifs. *Semin. Cell Dev. Biol.* **66**, 12–24.

Biology Open • Supplementary information
